# The Effects of Flavonoid Apigenin on Male Reproductive Health: Inhibition of Spermatogonial Proliferation through Downregulation of *Prmt7*/*Akt3* Pathway

**DOI:** 10.3390/ijms222212209

**Published:** 2021-11-11

**Authors:** Bingyuan Wang, Mingrui Zhang, Jiankang Guo, Zhiguo Liu, Rong Zhou, Fei Guo, Kui Li, Yulian Mu

**Affiliations:** 1 Institute of Animal Sciences, Chinese Academy of Agricultural Sciences, Beijing 100193, China; wangbingyuan@caas.cn (B.W.); ZMRSTC@163.com (M.Z.); g398829990@163.com (J.G.); liuzhiguo@caas.cn (Z.L.); zhourong03@caas.cn (R.Z.); 2 College of Animal Science and Technology, China Agricultural University, Beijing 100193, China; naoto86@163.com; 3 Agricultural Genomes Institute at Shenzhen, Chinese Academy of Agricultural Sciences, Shenzhen 518120, China

**Keywords:** apigenin, spermatogonial proliferation, *Prmt7*, *Akt3*, mouse

## Abstract

Apigenin, a common dietary flavonoid abundantly present in a variety of fruits and vegetables, has promising anticancer properties. As an effector of apigenin in myoblasts, protein arginine methyltransferase 7 (*Prmt7*) is required for male germ cell development. However, whether apigenin may influence male reproductive health through *Prmt7* is still unclear. To this end, mouse spermatogonia were treated with different concentrations (2.5 to 50 μM) of apigenin for 48 h, which showed that apigenin could cause reduced cell proliferation in conjunction with longer S phase and G2/M phase (with concentrations of 10 and 20 μM, respectively), and increased apoptosis of spermatogonia (with concentration of 20 μM). Reduced *Prmt7* expression was found in 20 μM apigenin-treated spermatogonia. Moreover, siRNA-induced *Prmt7* knockdown exhibited similar influence on spermatogonia as that of apigenin treatment. In mechanistic terms, transcriptome analysis revealed 287 differentially expressed genes between *Prmt7*-downregulated and control spermatogonia. Furthermore, rescue experiments suggested that the effects of apigenin on spermatogonia might be mediated through the *Prmt7*/*Akt3* pathway. Overall, our study supports that apigenin can interfere with mouse spermatogonial proliferation by way of the downregulated *Prmt7*/*Akt3* pathway, which demonstrates that the concentration should be taken into account in future applications of apigenin for cancer therapy of men.

## 1. Introduction

Infertility is a global public health issue and a major clinical concern. It is estimated that 8–12% of couples in reproductive age experience infertility worldwide and half of infertility cases are reportedly due to a male factor [1]. A meta-analysis discovered that sperm counts declined by 50–60% within four decades in industrialized countries. Further systematic review as well as meta-regression analysis showed that both sperm concentration and total sperm count exhibited declined trends within four decades [2,3,4]. The causes of infertility are multifactorial, among which nutrition has a significant impact on men’s reproductive health. For example, diets rich in processed meat, soy foods, potatoes, full-fat dairy and total dairy products, cheese, coffee, alcohol, sugar-sweetened beverages and sweets have been associated with detrimental effects on the quality of semen [5]. Several dietary components and nutrients have been considered as possible determinants of sperm function, fertility, or normal function of the reproductive system [6,7]. Therefore, identifying the dietary components or nutrients that influence male fertility is of great importance for the preservation of fertility, and this is even more so in those cases where fertility is threatened by, e.g., cancer treatment.

Apigenin has promising anticancer properties. Known chemically as 4′,5,7-trihydroxyflavone, apigenin is a common dietary flavonoid abundantly present in a variety of fruits and vegetables. It is one of the most abundant and most studied flavonoids, with molecular formula C_15_H_10_O_5_ [8]. So far, numerous studies have shown that apigenin has potential antioxidant, anti-inflammatory, antibacterial, antiviral, and anticancer properties. Among these properties, the anticancer roles of apigenin have been studied in more than 20 types of cancer. Results suggested that apigenin had selective cytotoxicity on cancer cells while sparing the normal cells from negative consequences [8,9,10]. Therefore, apigenin has been considered as a possible chemotherapeutic agent for cancer therapy. However, it is not clear if apigenin fights cancer while preserving physiological functions, such as reproduction. Apigenin protected testicular cells from acrylonitrile-induced inflammation and apoptosis [11] and sperm from acrylonitrile-induced subchronic injury [12], thereby suggesting that apigenin might be safe for the male germ cells. However, direct proof is missing. Effects of apigenin on male germ cells and its underlying molecular mechanisms are still unclear, and this impedes progress in the applications of apigenin.

It is important to review the mode of action of apigenin in other tissues, in order to infer how it might operate in male germ cells and design meaningful experiments. The mode of action is not entirely clear, but it involves the protein arginine methyltransferase 7 (*Prmt7*) in the skeletal muscle based on the fact that apigenin was reported to regulate muscle hypertrophy and myogenic differentiation through *Prmt7*-mediated pathways [13]. This is a member of the type III protein arginine methyltransferase family and the only member of this family that catalyzes monomethylated arginine residues on substrate proteins [14,15]. *Prmt7* knockout mice showed reduced body weight, increased fat mass, and less skeletal muscle mass, suggesting its function in muscle development and adipogenesis [16,17]. Similarly, PRMT7 deficient patients exhibited mild intellectual disability, obesity, symmetrical shortening of the digits, posterior metacarpals, and metatarsals, also implying its function in the development of skeletal muscle, neurons, bone, and the adipogenesis [17,18,19,20,21]. Further studies found that *Prmt7* was abundantly expressed in male mouse germ cells, including gonocytes and spermatogonia [22]. Depletion of *Prmt7* resulted in a defect of primordial germ cell proliferation during mouse embryonic stage, indicating that *Prmt7* is also involved in male germ cell proliferation [23]. It has been reported that the addition of apigenin to mouse diet led to quadriceps muscle weight increase in a dose-dependent manner and the muscle fiber size was also increased. Molecular mechanism analyses revealed that apigenin increased the expression of muscle mass regulator *Prmt7*, which promoted skeletal muscle hypertrophy and myogenic differentiation through *Prmt7*-*PGC-1α*-*GPR56* pathway and *Prmt7*-*p38*-*MyoD* pathway [13].

The purpose of this study was to investigate the effects of apigenin on spermatogonial proliferation, as well as the possible underlying molecular mechanisms, using a mouse spermatogonial cell line as in vitro model. Given the background knowledge on the interplay between apigenin and *Prmt7* in other tissues, we focused our experiments on *Prmt7*, as a candidate molecular mediator of the effects of apigenin in spermatogonia. Therefore, we hypothesized that apigenin may regulate spermatogonial proliferation through the *Prmt7*-meidated pathway. Our results revealed that certain concentrations of apigenin interfere with mouse spermatogonial proliferation by way of the downregulated *Prmt7*/*Akt3* pathway, which provides information useful for the dietary application as well as male cancer therapy of apigenin.

## 2. Results

### 2.1. Apigenin Hampers the Proliferation of Spermatogonia

**Figure 1:**
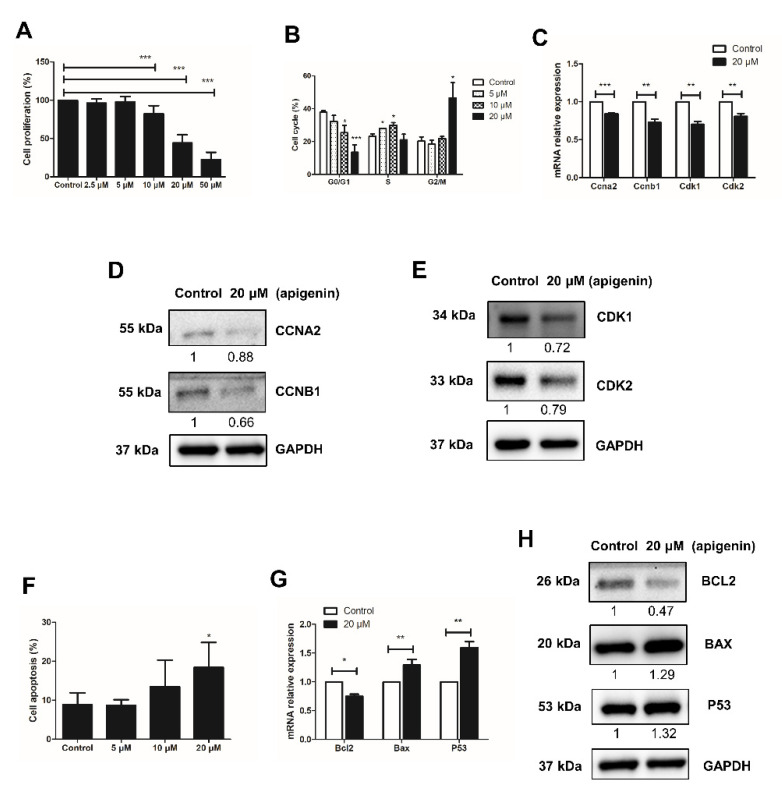
Effects of different concentrations of apigenin treatment on the proliferation and apoptosis of spermatogonia. (**A**) Cell proliferation was determined by CCK-8 assay after different concentrations of apigenin treatment for 48 h. (**B**) Cell cycle was analyzed by flow cytometry after applying different concentrations of apigenin treatment for 48 h. (**C**) Relative mRNA expression of *Ccna2*, *Ccnb1*, *Cdk1*, and *Cdk2* examined by RT-qPCR in spermatogonia treated with 0 and 20 μM apigenin for 48 h. (**D**,**E**) The protein levels of CCNA2, CCNB1 (**D**), and CDK1 as well as CDK2 (**E**) were detected by Western blotting in spermatogonia treated with 0 and 20 μM apigenin for 48 h. (**F**) Cell apoptosis was analyzed by flow cytometry after applying different concentrations of apigenin for 48 h. (**G**) Relative mRNA expression of *Bcl2*, *Bax*, and *P53* examined by RT-qPCR in spermatogonia treated with 0 and 20 μM apigenin for 48 h. (**H**)The protein levels of BCL2, BAX, P53 were detected by Western blotting in spermatogonia treated with 0 and 20 μM apigenin for 48 h. Data from three independent experiments are expressed as mean ± SEM. * indicates statistical significance. * *p* < 0.05, ** *p* < 0.01, and *** *p* < 0.001.

To determine the effect on spermatogonial proliferation, different concentrations of apigenin (0, 2.5, 5, 10, 20, or 50 μM) were applied in culture for 48 h. Compared with the control group (0 μM), apigenin treatment with 2.5 μM or 5 μM had no effect, while 10, 20, or 50 μM significantly reduced the proliferation of spermatogonia (*p* < 0.001, Figure 1A). We therefore selected 0, 5, 10, and 20 μM apigenin treatment to assess the cell cycle progression of spermatogonia. As shown in Figure 1B, compared with the control group, 5 and 10 μM apigenin treatment increased the percentage of spermatogonia in S phase. Interestingly, 20 μM apigenin treatment blocked spermatogonia in G2/M phase. Since the cell cycle is regulated by cyclins and cyclin-dependent kinases (Cdks), we further investigated their expression in 20 μM apigenin-treated spermatogonia, compared with the control group. The results showed that the relative mRNA expression of *Ccna2*, *Ccnb1*, *Cdk1*, and *Cdk2* was significantly decreased (Figure 1C). Similarly, the protein levels of these cell cycle regulators were also decreased (Figure 1D,E). These findings account the reduced expression of cell cycle regulators *Ccna2*, *Ccnb1*, *Cdk1*, and *Cdk2* for the inhibition of spermatogonial proliferation.

### 2.2. Apigenin Promotes Apoptosis of Spermatogonia

Next to the inhibitory effect of apigenin on the proliferation of spermatogonia, we examined its effect on apoptosis, using Annexin V-PI staining followed by flow cytometry analysis. Compared to control, the apoptosis rate of spermatogonia treated with 5, 10, and 20 μM apigenin increased in a dose-dependent manner: from no effect of 5 μM apigenin, to slightly increased apoptosis rate with 10 μM apigenin, to significantly increased apoptosis with 20 μM apigenin (Figure 1D). Therefore, the expression of apoptosis regulators in spermatogonia was further examined in the presence or absence of 20 μM apigenin. At this concentration *Bcl2* expression decreased while *Bax* and *P53* expression increased (Figure 1E,F), indicating that apigenin affected the expression of apoptosis regulators and thereby increased the apoptotic death of spermatogonia.

### 2.3. Apigenin Reduces the Expression of Prmt7 and Akt3 in Spermatogonia

**Figure 2:**
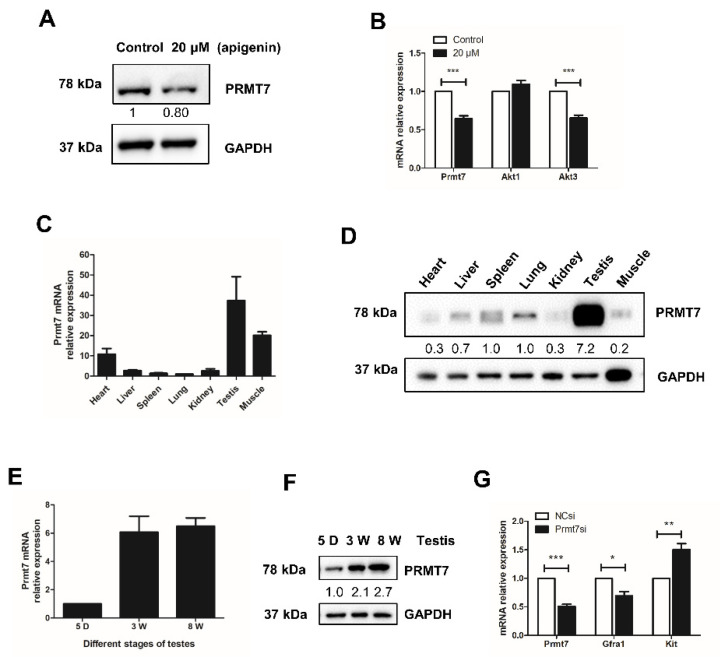
Effects of apigenin treatment on the expression of protein arginine methyltransferase 7 (*Prmt7*) and *Akt3* as well as *Prmt7* expression profile in mouse tissues. (**A**) The protein level of PRMT7 was detected by Western blotting in spermatogonia treated with 0 and 20 μM apigenin for 48 h. (**B**) Relative mRNA expression of *Prmt7*, *Akt1*, and *Akt3* was examined by RT-qPCR in spermatogonia treated with 0 and 20 μM apigenin for 48 h. (**C**) Relative mRNA expression of *Prmt7* examined by RT-qPCR in the heart, liver, spleen, lung, kidney, testis, and muscle of 8-week old mice. (**D**) The protein level of PRMT7 was detected by Western blotting in the heart, liver, spleen, lung, kidney, testis, and muscle of 8-week old mice. (**E**) Relative mRNA expression of *Prmt7* examined by RT-qPCR in the testes of 5-day (5 D), 3-week (3 W), and 8-week (8 W) old mice. (**F**) The protein level of PRMT7 was detected by Western blotting in the testes of 5 D, 3 W, and 8 W old mice. (**G**) Relative mRNA expression of *Prmt7*, *Gfra1*, and *Kit* examined by RT-qPCR in negative control siRNA (NCsi)- and *Prmt7* siRNA (Prmt7si)-transfected spermatogonia for 48 h. Data from three independent experiments are expressed as mean ± SEM. * indicates statistical significance. * *p* < 0.05, ** *p* < 0.01, and *** *p* < 0.001.

The effect of apigenin is probably mediated by multiple downstream effectors. Given the report that *Prmt7* and AKT pathways mediated the effect of apigenin on skeletal muscle hypertrophy and myogenic differentiation [13], we examined the effects of apigenin on the expression of *Prmt7* and AKT isoforms in spermatogonia. There are three isoforms of AKT, including *Akt1*, *Akt2*, and *Akt3*, among which *Akt3* initially was cloned from the rat brain and testis [24]. We found that 20 μM apigenin treatment decreased the expression of *Prmt7* on both mRNA and protein level (Figure 2A,B). The mRNA expression of *Akt1* was not affected, while the mRNA expression of *Akt3* was significantly decreased in spermatogonia treated with 20 μM apigenin (Figure 2B). These results indicated that 20 μM apigenin treatment inhibited the expression of *Prmt7* and *Akt3* in spermatogonia.

### 2.4. Prmt7 Is Abundantly Expressed in Mouse Testis

To further illuminate the molecular mechanism by which apigenin affects *Prmt7*-mediated spermatogonial proliferation, we profiled the expression of *Prmt7* in mouse tissues. Results showed that *Prmt7* was extensively expressed in heart, liver, spleen, lung, kidney, testis, and muscle of mouse. Notably, *Prmt7* was most abundant in testis (Figure 2C,D). Subsequently, we examined the expression of *Prmt7* in different stages of testicular maturation. As shown in Figure 2E,F, *Prmt7* expression increased during testicular maturation from 5-day, 3-week, to 8-week, underscoring its potential role in male reproduction. To corroborate this possibility, we examined the markers of spermatogonial stem cells after siRNA-mediated *Prmt7* knockdown. Results showed that *Prmt7* downregulation was accompanied by decreased expression of the spermatogonial stem cell marker *Gfra1*, and increased expression of the differentiation marker *Kit* (Figure 2G). These findings attested to the effect of *Prmt7* in spermatogonia.

### 2.5. Prmt7 Knockdown Recapitulates the Inhibitory Effects of Apigenin on Spermatogonial Proliferation

**Figure 3:**
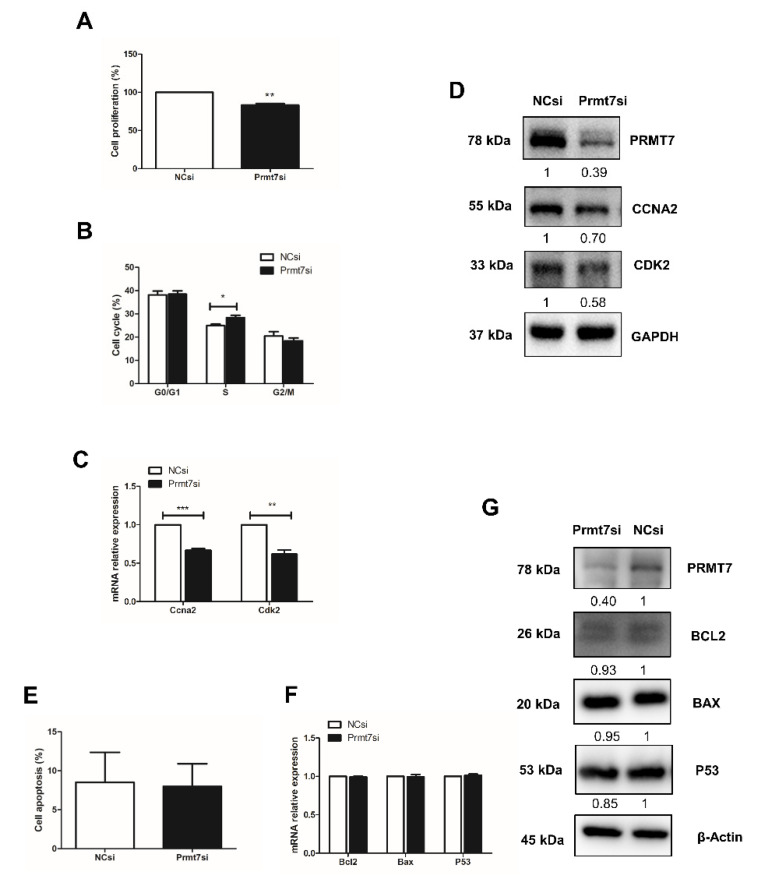
Effects of *Prmt7* knockdown on spermatogonial proliferation and apoptosis. (**A**) Cell proliferation was determined by CCK-8 assay after NC and *Prmt7* siRNA transfection into spermatogonia for 48 h. (**B**) Cell cycle was analyzed by flow cytometry after NC and *Prmt7* siRNA transfection into spermatogonia for 48 h. (**C**) Relative mRNA expression of *Ccna2* and *Cdk2* examined by RT-qPCR in spermatogonia transfected with NC and *Prmt7* siRNA for 48 h. (**D**) The protein level of PRMT7, CCNA2, and CDK2 detected by Western blotting in spermatogonia transfected with NC and *Prmt7* siRNA for 48 h. (**E**) Cell apoptosis was analyzed by flow cytometry after NC and *Prmt7* siRNA transfection into spermatogonia for 48 h. (**F**) Relative mRNA expression of *Bcl2*, *Bax*, and *P53* was examined by RT-qPCR in spermatogonia transfected with NC and *Prmt7* siRNA for 48 h. (**G**) The protein levels of PRMT7, BCL2, BAX, and P53 were detected by Western blotting in spermatogonia transfected with NC and *Prmt7* siRNA for 48 h. Data from three independent experiments are expressed as mean ± SEM. * indicates statistical significance. * *p* < 0.05, ** *p* < 0.01, and *** *p* < 0.001.

To directly test the role of *Prmt7* in spermatogonial proliferation, we measured the cell proliferation rate, the cell cycle progression, and the expression of cell cycle regulators after knockdown of *Prmt7*. After siRNA transfection for 48 h, the cell proliferation rate had declined (Figure 3A). Cell cycle analysis by flow cytometry revealed that the spermatogonia transfected with *Prmt7* siRNA were arrested in S phase (Figure 3B). The expression of cell cycle regulators *Ccna2* and *Cdk2* was decreased on both mRNA and protein level (Figure 3C,D). In addition, we performed Annexin V-PI staining followed by flow cytometry for apoptosis analysis, and examined the expression of apoptosis regulators *Bcl2*, *Bax*, and *P53* in *Prmt7*-knockdown spermatogonia. The results showed that downregulation of *Prmt7* had no significant effect on the cell apoptosis rate as well as the expression of *Bcl2*, *Bax*, and *P53*, neither on the mRNA nor on the protein level (Figure 3E–G). These findings demonstrated that *Prmt7* downregulation arrested spermatogonia in S phase and decreased spermatogonial proliferation rate, which is similar with the effects of apigenin in spermatogonia.

### 2.6. Downregulation of Prmt7 Expression Goes beyond This Gene and Perturbs Several Other Genes

**Figure 4:**
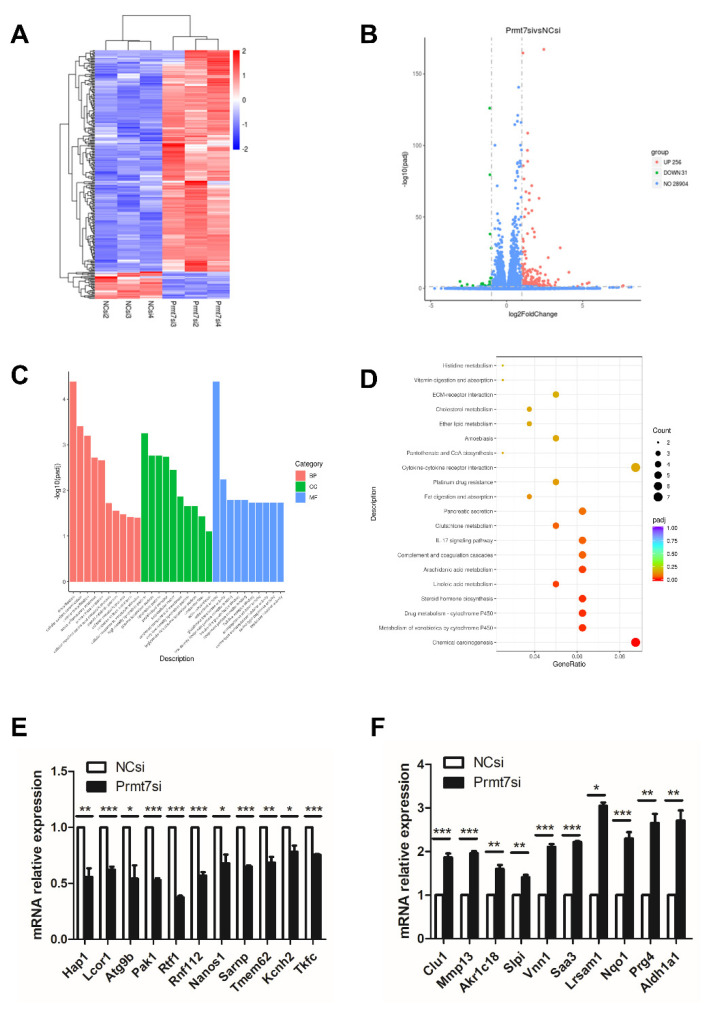
Transcriptome analysis (RNA sequencing) of spermatogonia transfected with NC and *Prmt7* siRNA. (**A**) Clustered heatmap of differentially expressed genes (DEGs) with criteria of adjusted *p* < 0.05, |log2 fold change| > = 1 was constructed according to the RNA expression level. Two groups with respective three replicates were classified. Blue represents low relative expression of RNAs, and red represents high relative expression of RNAs. (**B**) The volcano plot of 256 upregulated DEGs and 31 downregulated DEGs in Prmt7si-transfected spermatogonia relative to NCsi-transfected spermatogonia was shown. (**C**) The top 10 terms of biological process (BP), cellular component (CC), and molecular function (MF) of all DEGs with GO functional enrichment analysis were shown. (**D**) Top 20 pathways of all DEGs with KEGG pathway enrichment analysis were shown. (**E**) Verification of randomly selected downregulated DEGs by RT-qPCR. (**F**) Verification of randomly selected upregulated DEGs by RT-qPCR. Data from three independent experiments are expressed as mean ± SEM. * indicates statistical significance. * *p* < 0.05, ** *p* < 0.01, and *** *p* < 0.001.

To shed light on the possible molecular pathway underlying the similar effects of apigenin treatment and *Prmt7* knockdown in spermatogonia, we performed RNA sequencing analysis of *Prmt7* siRNA (Prmt7si)-transfected and NC siRNA (NCsi)-transfected spermatogonia, in triplicates. The Prmt7si and the NCsi groups are clearly resolved from each other in the cluster analysis (Figure 4A). In order to identify the differentially expressed genes (DEGs), we used the criteria of adjusted *p* < 0.05 and |log2 fold change| > = 1. As a result, a total of 287 DEGs were identified in the Prmt7si group compared to NCsi group (Figure 4B), including 256 upregulated DEGs and 31 downregulated DEGs. Next, the 287 DEGs were subjected to the GO functional enrichment analysis. The top 10 GO terms feature detoxification, cellular oxidant detoxification, cellular detoxification (biological process, BP), high-density lipoprotein particle, plasma lipoprotein particle, lipoprotein particle (cellular component, CC), and antioxidant activity, glutathione transferase activity, and low-density lipoprotein particle receptor binding (molecular function, MF), which are shown in Figure 4C. In addition, KEGG enrichment analysis of 287 DEGs revealed six significant pathways, including chemical carcinogenesis, metabolism of xenobiotics by cytochrome P450, drug metabolism-cytochrome P450, steroid hormone biosynthesis, linoleic acid metabolism, and arachidonic acid metabolism (Figure 4D). We checked the RNA sequencing results by RT-qPCR of randomly selected genes. These included 11 downregulated genes (*Hap1*, *Lcor1*, *Atg9b*, *Pak1*, *Rtf1*, *Rnf112*, *Nanos1*, *Sarnp*, *Tmem62*, *Kcnh2*, *Tkfc*) and 10 upregulated genes (*Clu1*, *Mmp13*, *Akr1c18*, *Slpi*, *Vnn1*, *Saa3*, *Lrsam1*, *Nqo1*, *Prg4*, *Aldh1a1*), which were confirmed by RT-qPCR (Figure 4E,F).

### 2.7. The Possible Functional Pathway Underlying the Regulation of Spermatogonial Proliferation

**Figure 5:**
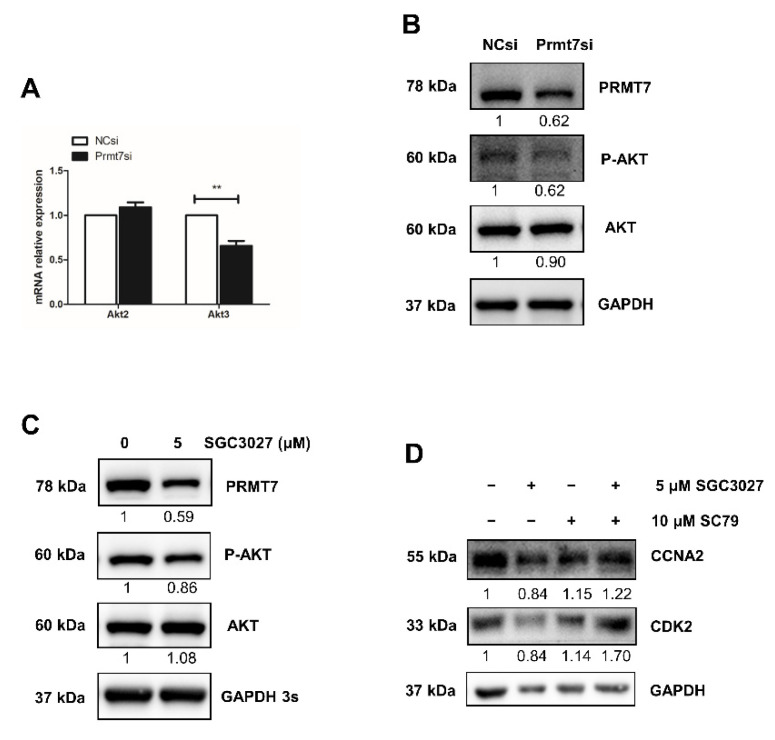
Apigenin/*Prmt7*/*Akt3* pathway analysis in spermatogonial proliferation. (**A**) Relative mRNA expression of *Akt2* and *Akt3* was examined by RT-qPCR in spermatogonia transfected with NC and *Prmt7* siRNA for 48 h. (**B**,**C**) The protein levels of PRMT7 and AKT as well as AKT phosphorylation (P-AKT) were detected by Western blotting in spermatogonia transfected with NC and *Prmt7* siRNA (**B**) or treated with 0 and 5 μM *Prmt7* inhibitor SGC3027 for 48 h (**C**). (**D**) The protein levels of CCNA2 and CDK2 were detected by Western blotting in spermatogonia treated with 5 μM *Prmt7* inhibitor SGC3027 and 10 μM AKT activator SC79 in different combination ways for the rescue assay. Data from three independent experiments are expressed as mean ± SEM. * indicates statistical significance. ** *p* < 0.01.

Among the *Prmt7*-regulated genes, we also found *Akt3*, the only AKT isoform identified in downregulated DEGs after *Prmt7* knockdown and featured also in the apigenin experiments. Therefore, we hypothesized that apigenin may regulate spermatogonial proliferation through *Prmt7*/*Akt3* pathway. The downregulation of *Akt3* (but not *Akt2*) in *Prmt7* siRNA-treated spermatogonia was confirmed by RT-qPCR (Figure 5A), lending further support to the results of the apigenin treatment. To ascertain the robustness of this result, we designed a rescue experiment, using SGC3027 (a potent and selective chemical inhibitor for *Prmt7*) and SC79 (a unique specific AKT activator). Both *Prmt7* siRNA-mediated *Prmt7* knockdown and 5 μM SGC3027 treatment-mediated *Prmt7* inhibition successfully resulted in decreased PRMT7 protein expression and AKT phosphorylation (Figure 5B,C). We next performed rescue experiments. As shown in Figure 5D, the decreased protein levels of cell cycle regulator CCNA2 and CDK2 in 5 μM SGC3027-treated spermatogonia can be partially rescued by the addition of 10 μM SC79. These results support that apigenin can regulate the proliferation of spermatogonia through the *Prmt7*/*Akt3* pathway.

## 3. Discussion

Here, we report that 20 μM apigenin hampered spermatogonial proliferation and induced spermatogonial apoptosis. This effect was mediated by the decreased expression of *Prmt7* and *Akt3*. Specifically, *Prmt7* knockdown suppressed spermatogonial proliferation, which could be attributed to the downregulated *Akt3* expression. Together, these findings converge on a mechanistic function of the apigenin-regulated *Prmt7*/*Akt3* pathway in mouse spermatogonia.

Studies have documented the anticancer effects of apigenin in more than 20 types of cancer cells [8,9,10]. These effects were mediated by suppressed cell proliferation, induced cell cycle arrest, and apoptosis. However, since natural flavonoids are ubiquitous, i.e., can be found in several dietary vegetables and fruits, it is conceivable that apigenin daily intake will also have an influence on physiological functions, e.g., male reproduction. It has been reported that the daily intake of apigenin ranged from 0.13–4.9 mg/day as the quantification of dietary intake of apigenin has large variation due to different geographical location, culture, and specific demographics [25,26]. Thus, in the current study, we supplemented different concentrations of apigenin (2.5–50 μM) in cultures of mouse spermatogonia, which covered the daily intake amount and anti-proliferative concentration in cancer cells, to test the direct role of apigenin in spermatogonial proliferation. As a result, apigenin at the concentrations of 10, 20, and 50 μM suppressed spermatogonial proliferation in a dose-dependent manner, arrested spermatogonia at S phase or G2/M phase, and also induced spermatogonial apoptosis. It has been reported that G2/M phase was arrested by 12.5, 25, and 50 μM apigenin treatment in colon cancer cells [27]. Apigenin induced both S and G2/M phase cell cycle arrest in combinational therapy of apigenin with gemcitabine in pancreatic cancer cells [28]. However, in human prostate cancer cells, treatment with apigenin at the concentration of 10 and 20 μM arrested cells at G0/G1 phase [29,30]. The discrepancy of the cell cycle arrest is possibly due to the cell type and apigenin concentration used. Nevertheless, similar to the effects in cancer cells, apigenin was able to suppress spermatogonial proliferation and increase their apoptosis, which was attributed to a decreased expression of cyclins and Cdks in conjunction with dysregulation of apoptosis-related factors.

Mice allowed ad libitum access to apigenin (standard diet containing 0.2% or 0.4% apigenin) showed increased PRMT7 protein expression in quadriceps muscle. Apigenin supplementation stimulated C2C12 myogenic differentiation with increased *Prmt7* expression and AKT phosphorylation [13]. However, in the current study, we discovered that apigenin treatment decreased *Prmt7* expression and AKT phosphorylation, which is at variance with the results obtained in the abovementioned study. As an anticancer candidate, apigenin inhibits cancer cells proliferation by downregulating AKT phosphorylation as well as metastasis by downregulating MMP-9 expression [31]. Similarly, *Prmt7* also played oncogenic roles in cancer cells by promoting cell proliferation and enhancing invasion [32,33]. These findings are consistent with the regulatory roles for *Prmt7* and AKT phosphorylation by apigenin. Given that *Prmt7* is an important muscle development regulator and an oncogene, the opposite effects of apigenin on the *Prmt7* expression and AKT phosphorylation might be attributed to their specific functions in specific conditions.

Given that apigenin treatment decreased *Prmt7* expression, we sought to determine whether apigenin affected spermatogonia by regulating *Prmt7*. We accomplished this by testing whether inhibiting *Prmt7* expression would have results similar to those of the treatment with apigenin, in spermatogonia. To this end, we transfected *Prmt7* siRNA to interfere *Prmt7* expression. Data showed that the proliferation of spermatogonia was suppressed and cell cycle was arrested at S phase after *Prmt7* downregulation. However, in the current study, *Prmt7* siRNA-mediated knockdown had no effect on spermatogonial apoptosis. When it comes to apigenin, 10 μM of concentration displayed similar results with that of *Prmt7* knockdown in spermatogonia. In addition, we found that *Prmt7* was highly expressed in mouse testis and *Prmt7* downregulation reduced *Gfra1* expression and increased *Kit* expression, spermatogonial stem cell marker and differentiation marker, respectively [34,35,36]. Therefore, the apigenin might function in spermatogonia by regulating *Prmt7* expression.

To further explore the molecular mechanisms of apigenin function in spermatogonial proliferation via *Prmt7*, we performed RNA sequencing in spermatogonia transfected with NC siRNA and *Prmt7* siRNA. The data displayed that the mRNA expression of *Prmt7*, *Ccna2*, *Ccnb1*, *Cdk1*, *Cdk2*, and *Akt3* was reduced (Table S2), which is consistent with the results from spermatogonia treated with apigenin or subjected to siRNA of *Prmt7*. It has been reported that *Akt3* is abundantly expressed in brain and testis. Although *Akt3* was reported involved in brain development, its functions still have not been well defined, including in male reproduction [37]. Noticeably, here, we found that only the expression of *Akt3*, but not other AKT isoforms, was downregulated in RNA sequencing data, which was further confirmed by RT-qPCR in spermatogonia treated with apigenin or *Prmt7* siRNA. Moreover, the decreased protein levels of cell cycle regulators after *Prmt7* inhibition can be partially rescued by the addition of AKT activator in spermatogonia. It has been reported that depletion of *Prmt7* resulted in a defect of primordial germ cell proliferation during mouse embryonic stage [23]. Hence, we assume that downregulated *Prmt7* expression will lead to early-stage germ cell loss. In addition, inhibiting *Akt3* leads to a cell cycle arrest of embryonic stem cells [38] and glial cell line-derived neurotrophic factor promoted spermatotonial stem cell self-renewal by blocking differentiation through increased *Akt3* [39], which implied the positive role of *Akt3* in the proliferation of spermatotonial stem cells. Therefore, we suppose that further downregulated *Akt3* expression arrested the spermatogonial stem cell proliferation but promoted its abnormal differentiation. These could lead to reduced sperm count and increased abnormal sperm count, consequently resulting in male infertility or subfertility. Overall, our findings suggested that the suppression of spermatogonial proliferation by apigenin treatment was mediated by the downregulated *Prmt7*/*Akt3* pathway and the concentration should be taken into account in future applications of apigenin for cancer therapy in men.

## 4. Materials and Methods

### 4.1. Animals and Chemicals

The 5 day-, 3 week-, and 8 week-old male ICR mice (at least 3 mice for each age) were purchased from Beijing Vital River. All animal experiments were performed in accordance with relevant institutional and national guidelines and regulations for the care and use of laboratory animals. The protocols and procedures employed were ethically reviewed and approved by the Animal Care and Use Committee in the Institute of Animal Sciences, Chinese Academy of Agricultural Sciences (Beijing, China) (ID: IAS2020-84). Tissue samples including heart, liver, spleen, lung, kidney, testis, and muscle were collected and immediately stored in liquid nitrogen until further processing. The *Prmt7* inhibitor SGC3027 (Cat. No.: HY-112445) and AKT activator SC79 (Cat. No.: HY-18749) were purchased from MedChemExpress, both of which were dissolved in dimethyl sulfoxide.

### 4.2. Cell Culture and Apigenin Treatment

Mouse GC-1 spermatogonial cell line was purchased from iCell (iCell-m022, Shanghai, China). Cells were cultured in high-glucose Dulbecco’s modified Eagle’s medium supplemented with 10% fetal bovine serum (Gibco, Amarillo, TX, USA), 100 U/mL penicillin and 100 μg/mL streptomycin (Invitrogen, Carlsbad, CA, USA) at 37 °C in a humidified incubator containing 5% CO_2_. Apigenin was purchased from Solarbio life sciences (IA0400). Different concentration of apigenin (0, 2.5, 5, 10, 20, or 50 μM) was supplemented into the culture medium after seeding the cells overnight. After treatment for 48 h, the cells were collected for further processing.

### 4.3. Cell Proliferation Assay

Cell proliferation was assessed by Cell Counting Kit 8 (CCK-8) (Dojindo, Kumamoto, Japan) according to the manufacturer’s instructions. Briefly, a total of 2500 cells/well were seeded into 96-well plates and cultured overnight. Next, the cells were treated with 0, 2.5, 5, 10, 20, or 50 μM apigenin addition or 50 nM siRNA transfection for 48 h. Thereafter, 10% of CCK-8 solution in culture medium was added into each well. The cells were further incubated at 37 °C for 1 h and the absorbance was measured by a microplate reader (Molecular Devices, SpectraMax M5, San Jose, CA, USA) at the wavelength of 450 nm. The cell viability (%) was calculated with the following equation: (Absorbance of experimental group–Absorbance of blank)/(Absorbance of control group–Absorbance of blank) * 100.

### 4.4. siRNA Transfection

siRNA transfection was performed using Lipofectamine RNAiMAX reagent (Invitrogen) according to the manufacturer’s instructions. Briefly, cells were seeded into 6-well plates at a density of 1 × 10^5^ cells/well one day before transfection and cultured in incubator with 5% CO_2_ at 37 °C. Later, 50 nM *Prmt7* siRNA or negative control (NC) siRNA was transfected into cells. After transfection for 48 h, the cells were collected for further analysis.

### 4.5. Cell Cycle Analysis by Flow Cytometry

After apigenin treatment or siRNA transfection, the spermagonia (1 × 10^6^ cells/mL) were collected and washed with PBS twice. Subsequently, the cells were stained with PI/RNase working solution prepared as manufacturer’s instructions (C543, Dojindo, Kumamoto, Japan) for 30 min at 4 °C in the dark. Thereafter, the cells were dispersed by vortexing, followed by incubation in fridge for 30 min at 4 °C. After another round of vortexing, the cells were filtered with 40 μm cell strainer to get single cells and analyzed on the flow cytometer within 1 h using FACSVerse flow cytometer (BD Biosciences, Franklin Lakes, NJ, USA).

### 4.6. Cell Apoptosis Analysis by Flow Cytometry

Spermatogonia treated with apigenin or siRNA transfection for 48 h were collected and washed with PBS twice. The preparation of cells using an apoptosis detection kit (AD10, Dojindo, Kumamoto, Japan) followed the manufacturer’s instructions. Briefly, the cells were digested and re-suspended in Annexin V binding buffer at a density of 1 × 10^6^ cells/mL. Then, 100 μL mixture was transferred into a new tube. Five microliter Annexin V-FITC and 5 μL PI were added into the mixture followed by incubation for 15 min at room temperature in the dark. Another 300 μL Annexin V binding buffer was added into the mixture for further flow cytometry within 1 h using FACSVerse flow cytometer (BD Biosciences).

### 4.7. RNA Extraction and RT-qPCR

Total RNA was extracted from mouse tissues and spermatogonia using TRIzol reagent (Invitrogen). The concentration of total RNA was measured with a Nano-100 spectrophotometer (Allsheng, Hangzhou, China) and a 260/280 ratio of ~2.0 was accepted as pure RNA. Then, using PrimeScriptTM RT reagent kit with gDNA eraser (TaKaRa, Cat. # RR047A), 1 μg RNA was reverse transcribed according to the manufacturer’s instructions. For RT-qPCR, a final volume of 20 μL reaction system contained 10 μL SYBR^®^ Premix Ex TaqTM (TaKaRa, Cat. # RR420A), 1 μL cDNA, 0.4 μL forward primer (work concentration 0.2 μM), 0.4 μL reverse primer (work concentration 0.2 μM), 0.4 μL ROX reference dye II, and 7.8 μL sterile distilled H_2_O was prepared. RT-qPCR was carried out on an ABI 7500 Fast Real-Time PCR system (Applied Biosystem, Waltham, MA, USA). Glyceraldehyde-3-phosphate dehydrogenase (GAPDH) was used as an internal reference gene. Relative mRNA expression was detected by normalizing gene expression against GAPDH expression using 2^-ddct^ method [40]. The primer sequences used for RT-qPCR are shown in Table S1. Results are presented as fold changes relative to the control groups.

### 4.8. Western Blotting

Mouse tissues and spermatogonia were lysed using protein extraction reagent containing protease and phosphatase inhibitor (Thermo Scientific, Waltham, MA, USA). The protein extracts were denatured, electrophoresed on 10% SDS-PAGE gel, and then transferred (blotted) to nitrocellulose membranes. The membranes were blocked with 5% nonfat milk for 1 h at room temperature and then incubated with primary antibodies overnight at 4 °C. The primary antibodies used were PRMT7 (D1K6R) Rabbit mAb (1:1000, #14762), Cyclin A2 (BF683) Mouse mAb (CCNA2, 1:1000, #4656), Cyclin B1 (D5C10) XP^®^ Rabbit mAb (CCNB1, 1:1000, #12231), cdc2 (E1Z6R) Rabbit mAb (CDK1, 1:1000, #28439), CDK2 (78B2) Rabbit mAb (1:1000, #2546), P53 (1C12) Mouse mAb (1:500, #2524), BCL2 (D17C4) Rabbit mAb (1:500, #3498), Phospho-Akt (Ser473) Antibody (P-AKT, 1:1000, #9271), β-Actin (13E5) Rabbit mAb (1:1000, #4970), GAPDH (14C10) Rabbit mAb (1:1000, #2118) from Cell Signaling Technologies, AKT Mouse mAb (1:2000, #60203-2-Ig) and BAX Mouse mAb (1:2000, #60267-1-Ig) from Proteintech. Later, the membranes were incubated with secondary antibody (Anti-rabbit IgG, HRP-linked antibody, 1:3000, #7074 or Anti-mouse IgG, HRP-linked antibody, 1:3000, #7076) for 1 h at room temperature. The blots were developed using Pierce ECL Western Blotting Substrate according to the manufacturer’s instructions (Pierce) and the protein bands were visualized on a Tanon-5200 Chemiluminescent Imaging System (Shanghai, China).

### 4.9. Library Preparation for RNA Sequencing

Total RNA was extracted from spermatogonia transfected with *Prmt7* siRNA or NC siRNA using TRIzol reagent (Invitrogen). A total amount of 1 μg RNA per sample was used as input material for the RNA library. Sequencing libraries for RNA were generated using NEBNext^®^ UltraTM RNA Library Prep Set for Illumina^®^ (New England Biolabs, Ipswich, MA, USA) following manufacturer’s protocols. Index codes were added to attribute sequences to each sample. The clustering of index-coded samples was performed on a cBot Cluster Generation System using TruSeq PE Cluster Kit v3-cBot-HS (Illumina, San Diego, CA, USA) according to the manufacturer’s instructions. After cluster generation, the library preparation was sequenced on an Illumina Novaseq platform and 150 bp paired-end reads were generated (Novogene, Beijing, China). The raw data of RNA sequencing are deposited in NCBI with accession numbers SAMN23039669, SAMN23039670, SAMN23039671, SAMN23039672, SAMN23039673, SAMN23039674.

### 4.10. RNA Sequencing Data Analysis

Clean reads were obtained after quality control from raw data of fastq format. After aligning the paired-end clean reads to the reference genome using Hisat2 v2.0.5, the featureCounts v1.5.0-p3 was used to count the reads number mapped to each gene. Differential expression analysis was performed using the DESeq2 R package (1.16.1). The resulting *p* values were adjusted using the Benjamini and Hochberg’s approach for controlling the false discovery rate. Genes with an adjusted *p* value < 0.05 were assigned as differentially expressed. Further, GO enrichment analysis of differentially expressed genes (DEGs) was implemented by clusterProfiler R package. GO terms with adjusted *p* value < 0.05 were considered significantly enriched. The statistical enrichment of DEGs in KEGG pathways (adjusted *p* value < 0.05) was tested using the clusterProfiler R package.

### 4.11. Statistical Analysis

The RT-qPCR data are presented as mean ± SEM from at least 3 independent experiments. Comparisons between two groups were analyzed using the Student’s t test. *p* <0.05 was considered statistically significant and indicated with asterisks (* *p* < 0.05; ** *p* < 0.01; *** *p* < 0.001).

## 5. Conclusions

In summary, our data support the concept that the anti-proliferative effects of apigenin on the spermatogonia are mediated through the downregulation of the *Prmt7*/*Akt3* pathway. Given the positive effects of apigenin as antioxidant, anti-inflammatory, antibacterial, antiviral, and anticancer supplement, our study raises awareness that apigenin might also have side-effects on male fertility, and this needs to be considered when apigenin is used for male cancer therapy.

## Data Availability

The data presented in this study are available on request from the corresponding author.

## Supplementary Materials

The following are available online at https://www.mdpi.com/article/10.3390/ijms222212209/s1.
