# Supplementary material for: The Effects of Flavonoid Apigenin on Male Reproductive Health: Inhibition of Spermatogonial Proliferation through Downregulation of Prmt7/Akt3 Pathway
Source: Int J Mol Sci. 2021 Nov 11;22(22):12209. doi: 10.3390/ijms222212209 (PMC8621337; doi:10.3390/ijms222212209)
Supplement: Supplementary file 1 [file ijms-22-12209-s001.zip › ijms-1434110-supplementary.pdf]

Table S1. The primers for RT-qPCR used in this study.

Gene name		Primer sequences
β-actin	F	TGTGCTGTCCCTGTATGCCTCT
	R	TAGATGGGCACAGTGTGGGTGA
Ccna2	F	TGGATGGCAGTTTTGAATCACC
	R	CCCTAAGGTACGTGTGAATGTC
Ccnb1	F	CAGGCAAGAGTGCCTCTGAA
	R	GTTTCCATCGGGCTTGGAGA
Cdk1	F	AAGTGTGGCCAGAAGTCGAG
	R	TCGTCCAGGTTCTTGACGTG
Cdk2	F	CGGAGTGGTGTACAAAGCCA
	R	TCGGATGGCAGTACTGGGTA
Bcl2	F	GAACTGGGGGAGGATTGTGG
	R	GCATGCTGGGGCCATATAGT
Bax	F	ATGGACGGGTCCGGGGAGCA
	R	CCCAGTTGAAGTTGCCGTCA
P53	F	CTCTCCCCCGCAAAAGAAAAA
	R	CGGAACATCTCGAAGCGTTTA
Prmt7	F	GGAGATTGCCAGGTCATCCT
	R	GAAGTCAGCCCCTGCAGTAA
Akt1	F	ATGAACGACGTAGCCATTGTG
	R	TTGTAGCCAATAAAGGTGCCAT
Akt2	F	ACGTGGTGAATACATCAAGACC
	R	CTACAGAGAAATTGTTCAGGGG
Akt3	F	ACCGCACACGTTTCTATGGT
	R	CGGCTCGGCCATAGTCATTA
Gfra1	F	AGGCTCAGAATTTGTTAATGG
	R	TAGGGCTCAAGGGAAGGAAG
Kit	F	GGCCTCACGAGTTCTATTTACG
	R	GGGGAGAGATTTCCCATCACAC
Ccne1	F	GCTTCGGGTCTGAGTTCCAA
	R	GTCTTGCAAAAACACGGCCA
Hap1	F	AGTTCATCACTGACCCTGCG
	R	GTTCTCTTCTGCCTGCCCAT
Lcor1	F	GCCTCATGCACTGTGTAGGT
	R	CTCCTCTGTTGGCGTTGACT
Atg9b	F	ACCCTTGCTTCATGTACCCG
	R	TAATCCACGCAGCGAAGGAG
Pak1	F	ACTATGATTGGAGCCGGCAG
	R	TGGCATTCCCGTAAACTCCC
Rtf1	F	CTCGGATACGGAAGACAGCG
	R	TTGCTCCCAAATGTCCACTCA

Continued Table S1

Gene name		Primer sequences
Rnf112	F	AGATGTTCGTTCACGTGGCT
	R	GTGCCCTTGCCCTGACTTAT
Nanos1	F	GCGCTCTACACCACACACAT
	R	TTTGGAGAGCGGGCAATACT
Sarnp	F	GGAGCTCCACAAGCTGAAG
	R	CCGATGCCATATCAACAGCC
Tmem62	F	TGCTGTTGGAGCACTACGG
	R	GATGTTGTTGGCTTCCGAGC
Kcnh2	F	TCACGTACTGTGACCTGCAC
	R	TGTGTCCTTGTCTGTACGCC
Tkfc	F	TGCTGGATCGGATATGCACC
	R	ACAATCCCCATCACCAGCAG
Clu1	F	ATCGGCTTCCCACACAAGAG
	R	CCCATAGTGGGAGGGAGACA
Mmp13	F	CTTCTGGCACACGCTTTTCC
	R	TGGCTTTTGCCAGTGTAGGT
Akr1c18	F	TAGGCCAGGCCATTCTAAGC
	R	CTCCATGGCCTTCAGAGACAC
Slpi	F	AAGCCACAATGCCGTACTGA
	R	CACACTGGTTTGCGAATGGG
Vnn1	F	TACTTTCCTCGCGGCTGTTT
	R	TGCTTCGCTGCAGATACGAT
Saa3	F	CAGCCAAAGATGGGTCCAGT
	R	CTCCGGGCAGCATCATAGTT
Lrsam1	F	GATGCATCGGCAGATCAGGA
	R	TCCTGCTTCGTTTCGCTCTT
Nqo1	F	CATTGCAGTGGTTTGGGGTG
	R	TCTGGAAAGGACCGTTGTCG
Prg4	F	CGGGCTGGATAATGCTATTCC
	R	CTAATGTCCCATTGCGCAGC
Aldh1a1	F	GGCCAAATCATCCCCTGGAA
	R	ATGTTTACCACGCCAGGAGG

Table S2. Gene expression in RNA sequencing data.

Gene Name	Gene ID	log2 Fold Change	Adjusted P
Prmt7	ENSMUSG00000060098	-0.762367163	1.49E-17
Ccna2	ENSMUSG00000027715	-0.398211242	3.34E-26
Ccnb1	ENSMUSG00000041431	-0.377923453	2.34E-24
Cdk1	ENSMUSG00000019942	-0.313085927	3.96E-17
Cdk2	ENSMUSG00000025358	-0.307024767	3.40E-10
Akt3	ENSMUSG00000019699	-0.144156259	0.043220681

Adjusted P < 0.05 is considered as significant difference.
